# Association of the androgens with COVID-19 prognostic outcomes: a systematic review

**DOI:** 10.1186/s13690-023-01168-2

**Published:** 2023-08-21

**Authors:** Linyu Zhang, Xinrui Sun, Ying Feng, Fang Ma

**Affiliations:** 1 Center for Translational Medicine, Key Laboratory of Birth Defects and Related Diseases of Women and Children (Sichuan University), Ministry of Education, West China Second University Hospital, Sichuan University, Chengdu, Sichuan 610041 P.R. China; 2 Department of Obstetrics and Gynecology, West China Second Hospital, Sichuan University, Chengdu, Sichuan 610041 P.R. China; 3 West China School of Basic Medical Sciences & Forensic Medicine, Sichuan University, Chengdu, Sichuan 610041 P.R. China

**Keywords:** Public health emergency, COVID-19, Androgens, Testosterone, Systematic review

## Abstract

**Objective:**

The coronavirus disease 2019 was a global public health emergency in later years (from 2020 to early 2022), and androgens have been associated with infection and prognostic outcomes. However, the relationship between low serum testosterone levels and prognostic outcomes remains inconclusive. This systematic review aimed to investigate the relationship between serum testosterone levels and prognostic outcomes in patients with COVID-19.

**Methods:**

We searched PubMed, MEDLINE, EMBASE and Web of Science electronic databases for all literature from January 1, 2020, to September 1, 2022. In addition, we also searched literature manually. The search terms were COVID-19, severe acute respiratory syndrome coronavirus 2 (SARS-CoV-2), androgens, and testosterone. There were no language restrictions for retrieval.

**Results:**

Our search identified 2285 articles, resulting in a full-text analysis of 16 studies, including 12 cohort studies and four case-control studies. Low serum testosterone levels were observed to be statistically associated with a higher probability of intensive care unit (ICU) admission in seven studies. In eight studies, higher hospital mortality was associated with lower serum testosterone levels. Six studies found that low serum testosterone levels were associated with a statistically significant difference in lung function impairment. Only four studies found that among living patients, those with lower serum testosterone levels had longer hospital stays. All but one of the included studies had a low risk of bias.

**Conclusions:**

Based on available data, low serum testosterone levels are associated with higher rates of ICU admission, hospital mortality, risk of lung failure, inflammatory markers, and longer hospital stays in patients with COVID-19 compared with those having normal serum testosterone levels.

## Introduction

The novel coronavirus disease 2019 has rapidly spread globally, causing great impacts on human health and the social economy. The severe acute respiratory syndrome coronavirus 2 (SASR-CoV-2) continues to mutate, resulting in a continued increase in the number of patients with COVID-19 worldwide. As of September 13, 2022, more than 60 million confirmed cases of COVID-19 have been reported to WHO, including more than 6 million deaths [1].

A study has identified more number of male patients with COVID-19 than female patients, which could be due to differences in sex hormone [2]. Androgens can up-regulate the activity of transmembrane serine protease 2 (TMPRSS2), which is required for the initiation of infection by SARS-CoV-2 [3] by promoting the interaction between the receptor-binding domain of the S1 subunit on SASR-CoV-2 spike glycoprotein and the angiotensin-converting enzyme 2 (ACE2) ectodomain and causing SARS-CoV-2 entry into human cells [4].

However, some studies have found that reduced serum testosterone levels are associated with a poor COVID-19 prognosis [5]. Chen et al. used publicly available gene expression datasets to show that ACE2 expression is reduced in hypoandrogen conditions [6]. This indicates that low serum testosterone levels are positively correlated with ACE2 levels. In addition, studies have shown that low ACE2 levels can aggravate lung damage in patients. In a mouse model of acid aspiration and sepsis-induced acute respiratory distress syndrome (ARDS), ACE2-deficient mice were found to have worsened oxygenation, massive pulmonary edema, increased infiltration of inflammatory factors, and hyaline membrane formation [7]. Consequently, an acute lung injury can also lead to decreased ACE2 expression and increased angiotensin II production [7, 8]. Testosterone, the major androgen, is involved not only in the reproductive system, but also in the motor system, endocrine system, blood system, and neuropsychiatric system [9]. Many studies have investigated the relationship between low serum testosterone levels and adverse prognostic outcomes, such as lung function impairment, increased length of hospital stay, intensive care unit (ICU) admission, mortality, and others in patients with COVID-19; however, the conclusions are contradictory. Most studies have shown that low serum testosterone levels are associated with higher ICU admission rates in patients with COVID-19, [10–16] whereas Karkin et al. found that patients admitted to the ICU had higher serum testosterone levels than those not admitted to the ICU [17]. Apaydin et al. also demonstrated that low serum testosterone levels were not associated with a poor prognosis of patients with COVID-19 [18].

Hence, to explore the relationship between serum testosterone levels and prognostic outcomes, such as lung function impairment, length of stay, ICU admission, and mortality in patients with COVID-19, we conducted a systematic review of the current evidence.

## Materials and methods

### Literature search strategy

The systematic review was conducted following the Preferred Reporting Items for Systematic Reviews and Meta-Analyses (PRISMA) guidelines [19], and the protocol has been registered on the PROSPERO platform. We searched PubMed, MEDLINE, EMBASE, and Web of Science electronic databases from January 1, 2020, to September 12, 2022. In addition, we manually searched the reference list of the relevant articles. The search terms were COVID-19, SASR-CoV-2, androgens, and testosterone (Table 1). During the search we did not consider language restrictions.

**Table 1:** Electronic search strategy

Database	Search term	Number
PubMed (All fields)	#1: COVID-19 OR SASR-CoV-2	#1: 328,411
	#2: androgens OR testosterone	#2: 18,923
	#3: #1 AND #2	#3: 453
Embase (All fields)	#1: COVID-19 OR SASR-CoV-2	#1: 306,520
	#2: androgens OR testosterone	#2: 35,186
	#3: #1 AND #2	#3: 713
Medline (All fields)	#1: COVID-19 OR SASR-CoV-2	#1: 201,191
	#2: androgens OR testosterone	#2: 12,234
	#3: #1 AND #2	#3: 658
Web of Science (Topic)	#1: COVID-19 OR SASR-CoV-2	#1: 360,122
	#2: androgens OR testosterone	#2: 23,421
	#3: #1 AND #2	#3: 457

### Eligibility criteria

This systematic review included all reported differences in serum testosterone levels between patients with COVID-19 and no COVID-19 in context to prognostic outcomes. The relationship between differences in serum testosterone levels and prognostic outcomes in patients with different severity of COVID-19 disease was also included. Studies were included if they reported any of the following measurements: ICU admission, hospital mortality, impairment of lung function, hospitalization time, and inflammation-related indicators. If the literature included data on testosterone levels in both men and women, we extracted data related to male testosterone and its prognosis only. In addition, we considered that all selected studies should have a control group.

Conference abstracts, editorials, reviews, case reports, duplicate publications, animal studies, no control arm, and intervention studies were excluded.

### Study selection and data extraction

Two authors independently searched the electronic database, screened the title and abstract of the retrieved literature according to the inclusion and exclusion criteria, and then conducted a full-text analysis of the eligible literature. Two researchers independently analyzed the full text and made decisions on inclusion. In case of disagreement, a discussion was conducted with the third author to solve the problem.

In cases of missing or incorrect data in the literature, we contacted the authors of the literature. Two researchers independently performed data extraction. Data were extracted for the first author, year, country, study design, sample size, age, population, characteristics of groups, blood test index, and outcomes. Other authors reviewed the final data extraction tables.

### Evaluation of the quality of research

We use Newcastle-Ottawa Score (NOS) to evaluate the quality of the included cohort studies in terms of selection, comparability, and outcome, and case-control studies in terms of selection, comparability, and exposure [20]. The differences between the two authors were discussed and resolved with the third author. NOS considers a score of six or above moderate to high in research quality and credibility.

## Results

### Literature search

**Fig. 1:**
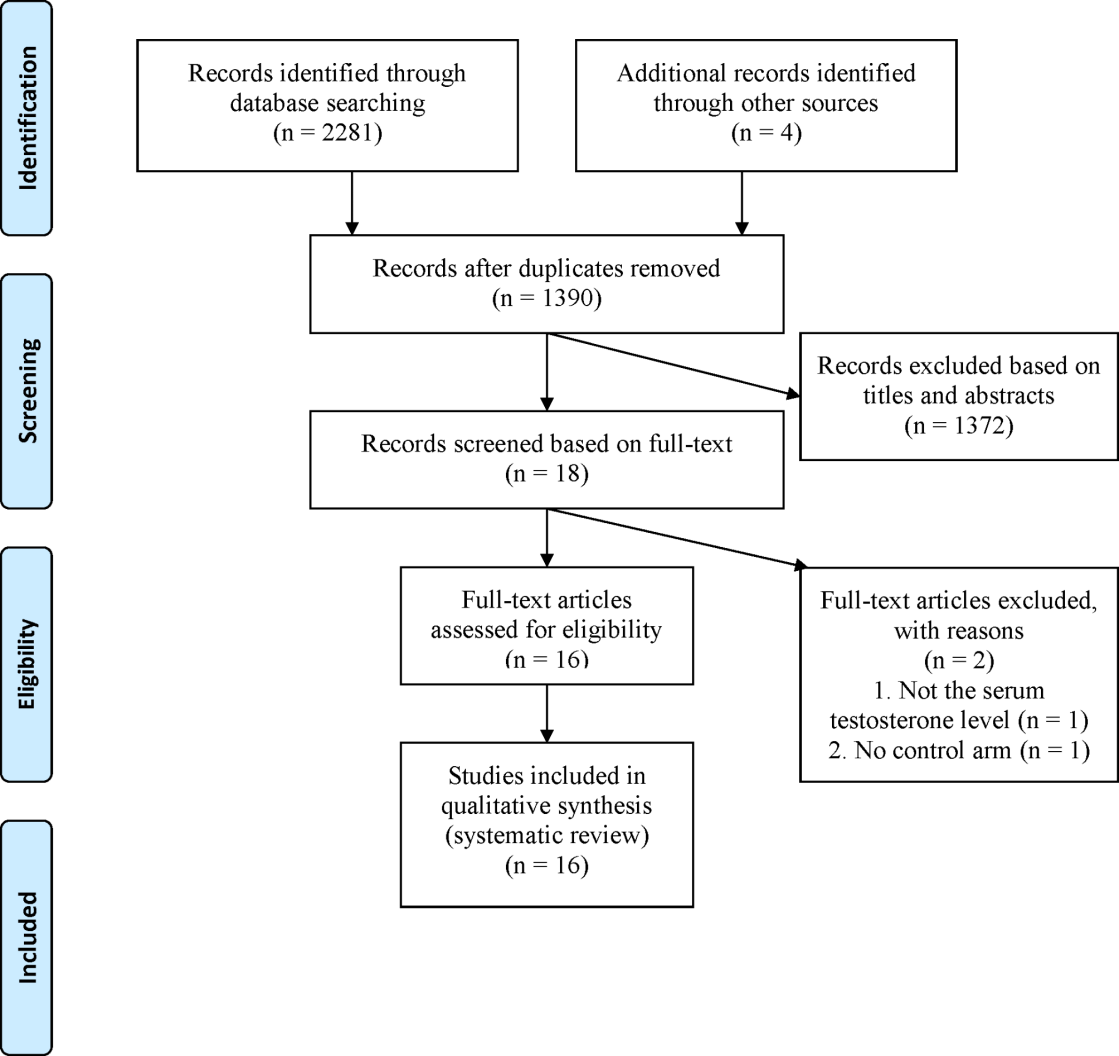
PRISMA flow diagram

A total of 2281 articles were retrieved from the four major databases, and four were retrieved manually, resulting in a total of 2285 articles. Of these, 895 duplicate articles were excluded. After evaluating the titles and abstracts of the remaining articles, 18 articles were found to be in line with the research purpose of the present study. After evaluating full-text of these 18 articles, two were excluded due to lack of the serum testosterone level data or control arm; hence, 16 articles were finally retained [10–18, 21–27]. Figure 1 shows the literature selection process.

### Patient characteristics

Table 2 presents the basic information of the selected articles. All the protocols were observational and included studies from seven countries, including Italy, Turkey, Spain, the Netherlands, Germany, China, and Australia. In total, studies involved 162,677 patients (mostly men between the age of 18 and 91 years). The studies were divided into groups based on serum testosterone levels, presence or absence of COVID-19, disease severity, and disease outcome. The blood test index included sex hormones and markers of inflammation. Outcomes included pulmonary complications associated with poor prognosis, ICU admission, length of hospital stay, and death.

**Table 2:** Study characteristics and outcomes

First author, year	Country	Study design	Sample size	Age, years	Population	BMI groups and number of cycles	Blood test index	Outcomes
Çayan, 2020	Turkey	prospective cohort study	438	(43–57)	COVID-19 male and female patients	divided into three groups according to asymptomatic, symptomatic IMU hospitalization, and ICU hospitalization.	TT, FSH, LH, prolactin, estrogen; biochemical indicators	ICU admission, and length of hospital stay
Salciccia, 2020	Italy	cohort study	29	(23–90)	COVID-19 male patients	Groups were assigned according to the need for invasive oxygen assistance.	Complete blood count; TT; biochemical indicators	Invasive oxygen assistance
Camici, 2021	Italy	case-control study	48	(18–65)	COVID-19 male patients	24 patients with severe pneumonia in case group; mild COVID-19 in control group	Sex hormone on admission; Markers of systemic inflammation	Presence of hyperinflammatory syndrome; Percentage of damaged lung volume; Length of hospital stay; Time to swab clearance; Time to death
Infante, 2021	Italy	case-control study	59	(38–89)	COVID-19 male patients	two groups: survivors and non-survivors.	TT, estrogen progesterone, prolactin	ICU admission; coagulation and Sepsis; hospital mortality
Karkin, 2021	Turkey	prospective cohort study	70	(20-over 60)	COVID-19 male patients	divided according to the need for intensive care	TT	ICU admission
Salciccia, 2021	Italy	cohort study	25	(41–78)	COVID-19 male patients	stratified according to impaired TT levels at the moment of hospital admission	TT; complete blood count; biochemical indicators	Viral positivity durations
Salonia, 2021	Italy	case-control study	567	(49–66)	COVID-19 male patients; healthy men	divided into four groups according to the severity of COVID-19	FSH, LH, TT, estrogen; biochemical indicators	ICU admission; death
Schroeder, 2021	Germany	retrospective cohort study	92	(58–82)	SARS-CoV-2 positive and negative patients	admitted to the Department of Intensive Care Medicine	TT	ARDS; mechanical ventilation; ICU stay
van Zeggeren, 2021	the Netherlands	case-control study	40	(63–78)	Amsterdam UMC COVID-19 Biobank patients	consisting of postmenopausal women and age-matched men, with a mortality of 50% in each group	estradiol; TT; SHBG	ICU admission; Invasive ventilation; Deceased
Apaydin, 2022	Turkey	cohort study	81	(22–60)	COVID-19 male patients	divided patients into low and normal testosterone groups	FSH; LH; SHBG; TT	Duration of hospitalization; ICU admission
Cinislioglu, 2022	Turkey	prospective cohort study	450	(25–91)	COVID-19 male patients; non-COVID-19 patients	divided into mild/moderate and severe groups	TT, FSH, LH	lung involvement; ICU admission; death
Marinelli, 2022	Italy	prospective, multicentric study	35	(58–74)	COVID-19 male patients	two groups: alive and dead	hormonal profile; inflammatory/biochemical profile	CPAP use; hospitalization time; in-hospital mortality rate
Toscano‑Guerra, 2022	Spain	retrospective cohort study	497	(56–71)	COVID-19 male and female patients	classified as mild, moderate, severe-survivor, and severe-deceased	TT; biochemical and hematological parameters	severity; death
Vena, 2022	Italy	cohort study	221	(26–93)	COVID-19 male patients	Subgroups by serum total testosterone values	TT	Respiratory outcomes; In‑hospital mortality
Yeap, 2022	Australia	prospective cohort study	159,964	(40–69)	community-dwelling adults	5558 were infected with SARS-CoV-2, with 438 deaths from COVID-19	TT; SHBG	Deaths related to COVID-19;
Zheng, 2022	China	cohort study	61	(37–74)	COVID-19 male patients	classified as ICU and non-ICU groups	TT; laboratory indicators	PSI; disease severity

ARDS, acute respiratory distress syndrome; COVID-19, coronavirus disease 2019; CPAP, continuous positive airway pressure; FSH, follicle-stimulating hormone; IMU, internal medicine unit; ICU, intensive care unit; LH, luteinizing hormone; PSI, pneumonia severity index; SARS-CoV-2, severe acute respiratory syndrome coronavirus 2; SHBG, sex hormone binding globulin; TT, total testosterone

### Quality assessment

Except for one cohort study [16], for which the total quality assessment score was four, all the other cohort studies had a score of six or above. Hence, the overall quality of the cohort study was acceptable. Further, the total score of quality assessment of observational studies was seven or above, indicating that the overall quality was medium to high (Table 3).

**Table 3:** Outcome of assessment of the quality of non-randomized studies using the Newcastle-Ottawa scale

cohort studies	Selection				Comparability		Outcome			
	Representativeness of the exposed cohort	Selection of non-exposed cohort	Ascertainment of exposure	Outcome not presented at the start	age	Most of additional factors	Assessment of outcome	Follow-up long enough	Adequacy of follow up	Total score
Çayan, 2020	*	*	*	-	-	*	*	*	*	7/9
Salciccia, 2020	*	*	*	*	*	*	-	-	-	6/9
Karkin, 2021	*	*	*	-	-	*	*	*	-	6/9
Salciccia, 2021	*	*	*	-	*	*	-	*	-	6/9
Schroeder, 2021	*	*	*	-	*	*	*	*	*	8/9
Apaydin, 2022	*	*	*	*	*	*	-	*	*	8/9
Cinislioglu, 2022	*	*	*	*	*	*	-	*	*	8/9
Marinelli, 2022	*	*	*	-	*	*	*	*	-	7/9
Toscano Guerra, 2022	*	*	*	-	-	*	*	*	-	6/9
Vena, 2022	*	*	*	*	-	*	-	*	*	7/9
Yeap, 2022	*	*	*	-	-	*	*	*	*	7/9
Zheng, 2022	*	*	*	-	-	*	-	-	-	4/9
case-control	Selection				Comparability		Outcome			
	Is the case definition adequate?	Representativeness of the cases	Selection of Controls	Definition of Controls	age	Most of additional factors	Assessment of outcome	Same method of ascertainment for cases and controls	Non- Response rate	Total score
Camici, 2021	*	*	*	*	*	*	-	*	*	8/9
Infante, 2021	*	*	*	*	*	*	*	*	-	8/9
Salonia, 2021	*	*	*	*	-	*	-	*	*	7/9
van Zeggeren, 2021	*	*	*	*	*	*	*	*	*	9/9

A single asterisk (*) indicates 1 score, and dash (-) indicates 0 score

### Outcomes

#### ICU admission

Nine studies [10–18] evaluated the relationship between serum total testosterone (TT) levels and ICU admission rates, and eight of them found statistical significance. Çayan et al. found that the mean serum TT level was significantly lower in the ICU admission group than that in the non-ICU admission group (239.94 ± 187.88 vs. 326.73 ± 152.18, p = 0.001). The probability of ICU admission rate during hospitalization increased significantly with decreasing baseline serum TT levels (linear equation p = 0.001, logarithmic equation p = 0.000) [10]. Infante et al. found that compared with the survival group, the non-survival group had a lower serum TT level but a higher probability of ICU admission (79.4% vs. 35%, p < 0.0001) [11]. However, Karkin et al. suggested that serum TT levels were significantly higher in the intensive care need group than that of the non-intensive care need group (291 ng/dL [112–531] vs. 390 ng/dL [180–680], p < 0.05) [17]. In a univariate analysis by Salonia et al., serum TT levels were inversely associated with ICU admission (odds ratio [OR]: 0.54, 95% confidence interval [CI]: 0.43–0.67, p < 0.0001), whereas in multivariate analysis, serum TT levels were associated with the requirement of ICU admission (OR: 0.53, 95% CI: 0.39–0.74, p < 0.0001) [12]. Schroeder et al. discovered that critically ill male patients with COVID-19 admitted to the ICU had the lowest serum TT levels compared with critically ill male patients with no COVID-19 (p < 0.0001) [13]. However, Apaydin et al. found no significant difference in ICU admission rates between the low and normal serum TT level groups (20.6% vs. 4.3%, p = 0.071) [18]. Cinislioglu et al. found that serum TT levels were statistically significant in univariate and multivariate binary logistic regression analyses (OR: 0.985, 95% CI: 0.985–0.993) to assess the need for ICU admission [14]. Using OR estimation and logistic regression analysis to assess the risk of ICU admission in patients with a mild-moderate prognosis, Toscano-Guerra et al. discovered that serum TT levels are significant (OR: 0.17, 95% CI 0.09–0.31) [15]. Zheng et al. showed that serum TT levels in the non-ICU admission group were statistically significant compared with those in the ICU admission group (6.7 nmol/L [4.2–8.7] vs. 3.7 nmol/L [1.5–4.7], p = 0. 012) [16]. Taken together, most studies suggest that patients with low serum TT levels are at higher risk of being admitted to the ICU.

#### Hospital mortality

A total of 10 [10–12, 15, 18, 22, 24–27] studies reported an association between serum TT levels and mortality. Çayan et al. found that the probability of mortality increased significantly with a decrease in baseline serum TT levels (linear equation p = 0.002, logarithmic equation p = 0.000). In univariate analysis, reduced serum TT level (OR: 1.008) was a highly significant predictor of death in patients with COVID-19 [10]. However, Camici et al. discovered no association between serum TT levels and risk of death (per 1 ng/mL TT, hazard ratio [HR]: 0.49, 95% CI 0.19–1.28, p = 0.147) [22]. Infante et al. showed significantly lower serum TT levels in the non-survivor group compared with the survivor group (< 100 ng/ dL) (71.8% vs. 35%, p < 0.0001) [11]. Salonia et al. used univariate analysis to show that serum TT levels were negatively associated with mortality outcomes (OR: 0.68, 95% CI: 0.53–0.86, p < 0.002). Furthermore, in multivariate analysis, serum TT levels were associated with mortality outcomes (OR: 0.67, 95% CI: 0.46–0.99, p < 0.05) [12]. van Zeggeren et al. found that serum TT levels were lower in dead patients compared with those who survived (0.8 [interquartile range, ITR 0.4–1.9), 3.2 (IQR 2.1–7.5), p < 0.001] [24]. Although dead patients in the Apaydin et al. study had very low free testosterone (FT) levels (3.13 µg/L, 5.37 µg/L, and 6.21 µg/L, respectively), no between-group comparisons were performed because there were only three patient [18]. In the study by Marinelli et al., serum TT and calculated FT levels were lower in dead patients [TT 1.55 ng/mL (0.77–2.29) vs. 1.98 ng/mL (1.30–2.72)]; calculated FT [0.0222 ng/mL (0.0134–0.0703) vs. 0.0441 ng/mL (0.0256–0.0742)] but the difference was not statistically significant [25]. The receiver operating characteristic (ROC) curve and area under the ROC curve (AUC) values calculated by Toscano-Guerra et al., based on the slope of the trajectory of the serum TT level curve, showed that all survivors were compared with those who died (AUC = 0.9281, 95% CI 0.8801–0.9761, p < 0.0001), and severe survivors vs. dead patients (AUC = 0.9205, 95% CI 0.8664–0.9747, p < 0.0001), indicating that the serum testosterone trajectory is a very accurate predictor of COVID-19 survival [15]. Vena et al. showed that in a univariate regression model, in-hospital mortality was associated with serum TT levels (OR: 0.75, 95% CI: 0.64–0.87, p < 0.001). Further, in multivariate regression analysis, low serum TT levels remained significantly associated with in-hospital mortality (OR: 0.68, 95% CI 0.52–0.88, p = 0.004) [26]. Yeap et al. showed an association between serum TT levels and the risk of death from COVID-19 in a fully adjusted model, including exposure variables, age, region, and region/time interaction, a full set of sociodemographic characteristics, lifestyle, medical variables, and time of blood collection (overall trend, p = 0.008) [27]. In summary, most findings suggest that serum TT levels are negatively associated with hospital mortality in patients with COVID-19.

#### Impairment of lung function

The association between serum TT levels and lung injury in patients with COVID-19 was statistically significant in six studies [16, 21–23, 25, 26]. After adjusting for age-adjusted Charlson Comorbidity Index, history of hypertension, dyslipidemia, and smoking status, Salciccia et al. found that a higher serum TT level was independently associated with a lower odd of invasive oxygenation (OR: 0.43, 95% CI: 0.23–0.85, p = 0.016) [21]. Camici et al. showed lower serum TT, calculated FT, and bioavailable testosterone (BioT) levels in severe pneumonia cases compared with mild COVID-19 (1.4 ng/mL [IQR 0.7–2.8] vs. 3.5 ng/mL [IQR 2.2–4.2], p = 0.005] [22]. In the Salciccia et al. study, patients requiring invasive oxygen support on admission were associated with lower median serum TT levels (2.64 ng/mL [IQR 1.56–2.99] vs. 5.4 ng/mL [IQR 4.47–5.56], p = 0.011] [23]. After adjusting for age and oxygenation index (PaO_2_/FiO_2_) [16], Marinelli et al. showed that serum TT and calculated FT levels were negatively correlated with continuous positive airway pressure use in multivariate logistic regression analysis (OR: 0.109, 95% CI: 0.0129–0.916, p < 0.001) [25]. Vena et al. found that the group with normal serum TT levels (T > 12 nmol/L) in comparison with the lower serum TT levels group (T < 8 nmol/L) had significantly higher PaO_2_/FiO_2_ ratio (278.1 [97.0–592.0] vs. 328.0 [247.0–452.0], p = 0.001] [26]. Zheng et al. used Spearman correlation analysis to evaluate the relationship between serum TT level and pneumonia severity index (PSI) and showed that the serum TT level was negatively correlated with PSI in both the first and second week after the onset of COVID-19 (r = -0.372, p = 0.043 and r = -0.502, p < 0.001, respectively) [16]. All relevant studies indicated that patients with low serum TT levels had a more severe impairment of the lung function.

#### Hospitalization time

Only five studies [11, 14, 18, 22, 25] demonstrated the relationship between serum TT level and length of hospital stay, and the differences were statistically significant. Camici et al. showed that TT, calculated FT, and BioT values were negatively correlated with length of hospital stay (p = 0.052, p = 0.041, and p = 0.023, respectively) [22]. Infante et al. found that although the non-survivor group had a significantly higher proportion of patients with severely low serum TT levels compared with survivors (71.8% vs. 35%, p < 0.0001), the mean length of hospital stay was significantly longer in the survivor group than that in the non-survivor group (34 ± 19 days vs. 14 ± 7 days, p < 0.0001) [11]. Correlation analysis by Apaydin et al. showed that FT level was negatively correlated with length of hospital stay (ρ = 0.334, p = 0.002) [18]. Cinislioglu et al. also showed a significant negative correlation between serum TT level and length of hospital stay (r = − 0.316, p < 0.001) [14]. Marinelli et al. showed that in the surviving subgroup, a longer hospital stay was significantly associated with lower serum TT levels and calculated FT levels (ρ = 0.51, p < 0.01 and ρ = 0.55, p < 0.01) [25]. Hence, four studies showed a negative correlation between serum TT levels and hospitalization time.

#### Inflammation-related indicators

The association between serum TT levels and inflammatory markers was described in seven studies [11, 14–16, 18, 21, 22]. Salciccia et al. showed that serum TT level was significantly negatively correlated with C-reactive protein (CRP), pH, IL-6, and D-dimer (ρ = -0.601, p = 0.001; ρ = -0.446, p = 0.037; ρ = -0.670, p = 0.001; ρ = -0.434, p = 0.049, respectively). Notably, a significant positive association was established between serum TT levels and monocytes (ρ = 0.482, p = 0.017), whereas no further significant associations were observed for other leukocyte lines [21]. In the study by Camici et al., patients with the hyperinflammatory syndrome had significantly lower median serum TT levels than patients with lower inflammatory response (1.7 ng/mL [IQR 0.80–2.60] vs. 3.6 ng/mL [IQR 1.70–4.30], p = 0.018], and lower serum TT level was significantly associated with lower lymphocyte and higher neutrophil counts (ρ = 0.31, p = 0.038; ρ = 0.43, p = 0.003, respectively) [22]. Infante et al. showed a significant negative correlation between serum TT level and CRP, IL-6, and D-dimer (ρ = -0.350, p = 0.006; ρ = − 0.266, p = 0.04; ρ = − 0.327, p = 0.01, respectively), and negatively correlated with WBC count (ρ = -0.255, p = 0.05) [11]. In the study of Apaydin et al., FT level was positively correlated with lymphocyte count and lymphocyte percentage (ρ = 0.260, p = 0.018; ρ = 0.346, p = 0.001, respectively) [18]. Cinislioglu et al. also showed that serum TT level was significantly negatively correlated with serum D-dimer, lactate dehydrogenase, and CRP (r = − 0.278, p < 0.001; r = − 0.510, p < 0.001; r = − 0.527, p < 0.001, respectively) and significantly positively correlated with lymphocyte level (r = 0.515, p < 0.001) [14]. Toscano-guerra et al. also showed a significant correlation between serum TT level and lymphocyte count (absolute count, r = 0.3122; WBC score, r = 0.4187), and neutrophil count was also negatively correlated (r = -0.3586) [15]. At 2 weeks after onset, Zheng et al. found that serum testosterone level was positively correlated with lymphocyte count (r = 0.522, p < 0.05) and negatively correlated with the neutrophil count, LDH, CRP, and D-dimer (r = -0.358, -0.519, -0.403, -0.533, p < 0.05) [16]. In these studies, serum TT levels were negatively correlated with several classical inflammatory markers, while positively correlated with monocyte and lymphocyte counts.

## Discussion

This systematic review of 16 observational studies assessed the association between serum testosterone levels and outcomes in patients with COVID-19. This study showed that low serum testosterone levels were associated with higher rates of ICU admission, hospital mortality, risk of lung failure, inflammatory markers, and longer hospital stays in patients with COVID-19 in comparison to those with normal serum testosterone levels. These adverse outcomes may have long-term effects on patients with COVID-19. For example, ICU admission and prolonged hospital stay may increase the risk of the incidence of iatrogenic infections [28].

Our results have important implications for clinical work. Serum testosterone level may be used to predict the prognosis of patients with COVID-19 but existence of a causal relationship between serum testosterone and COVID-19 is yet to be determined.

Androgens are an important factor in the regulation of penile erection. However, we note conflicting points in the study by Karkin et al. where patients with severe COVID-19 had higher testosterone levels and lower erectile function scores compared with patients without severe COVID-19 [17]. In addition, in terms of length of hospital stay, Infante et al. found that although serum testosterone levels were higher in the survival group than that in the non-survival group, the mean length of hospital stay in the survival group was significantly longer than that in the non-survival group [11]. In contrast to other findings, on average, people who have died from severe COVID-19 are expected to have shorter hospital stays than those who survived. Hence, the association between lower serum testosterone levels and longer hospital stays occurred primarily in survivors. Included studies had inconsistent definitions of serum testosterone levels and used different units of measurement, which may also have contributed to the large heterogeneity among them. Moreover, inflammation is a more important indicator than personal characteristics and comorbidities in the admission population [29]. Independent associations were identified between inflammatory biomarkers and the need for respiratory support or mortality outcomes in patients with COVID-19 [30]. Our study also found an inverse relationship between serum TT levels and inflammatory biomarkers, such as CRP, IL-6, and D-dimer. Lymphocytes play a decisive role in maintaining systemic immune homeostasis and inflammation, and lymphopenia is a predictor of prognosis in patients with COVID-19 [31]. Our results showed a positive correlation between serum TT level and lymphocyte count, suggesting that low serum TT level may be a prognostic factor for COVID-19.

Androgens can regulate the enzyme activity of TMPRSS2, which promotes SASR-CoV-2 interaction with ACE2 in human cells [3]. However, ACE2 expression is reduced in the presence of hypoandrogen [6]. A study has demonstrated that low levels of ACE2 and high levels of angiotensin II may lead to pneumonia, resulting in increased pulmonary vascular permeability [32]. This may explain why low serum testosterone levels are associated with poor prognosis in COVID-19. In addition, Welen et al. found that anti-androgens do not benefit patients with COVID-19 and should not be used in hospitalized patients with COVID-19 or as a preventive measure for COVID-19 [33]. Although this study did not measure the subjects’ serum testosterone levels before anti-androgens therapy, it still suggests that low androgen levels are not a favorable factor for COVID-19. It should be noted that the studies included in this systematic review were observational and could only show that low serum testosterone levels were associated with poor prognosis in patients with COVID-19.

Our study has several limitations. First, it is not clear whether testosterone levels of analyzed patients were normal before they contracted COVID-19. Hence, a causal relationship between low testosterone and poor prognosis in men is yet to be explored. Second, the heterogeneity between the included cohorts and case-control studies was largely due to different types of studies and differences in population characteristics, which prevented us from conducting a summary analysis of the primary outcome data. In addition, these studies also differ in the judgment criteria of patient severity, the inclusion criteria of subjects, and the control protocol. In fact, most of the individual studies included in the systematic review did not measure SHBG (sex hormone binding globulin), which is a testosterone binding protein, but only provides TT levels. Therefore, high-level studies are needed to confirm whether intervention patients with serum testosterone levels can improve the outcome of patients with COVID-19.

## Conclusion

In conclusion, our findings suggest that low serum testosterone levels may be associated with higher ICU admission, mortality, risk of lung failure, inflammatory markers, and longer hospital stay in patients with COVID-19, and hence these admitted patients should receive more attention upon admission. Furthermore, the serum TT level in patients with COVID-19 can act as an indicator to predict the outcome and may even become an adjuvant therapy strategy to improve the outcome.

## Data Availability

No new data were generated or analyzed in support of this research.

## Abbreviations

ACE2: Angiotensin-converting enzyme 2
ARDS: Acute respiratory distress syndrome
BioT: Bioavailable testosterone
COVID-19: Coronavirus disease 2019
FT: Free testosterone
ICU: Intensive care unit
SARS-CoV-2: Severe acute respiratory syndrome coronavirus 2
SHBG: Sex hormone binding globulin
TMPRSS2: Transmembrane serine protease 2
TT: Total testosterone

## References

[1] World Health Organization Coronavirus Disease (Covid-19) Dashboard. 2023 February 22, 2023 February 22, 2023]; Available from: Available at: https://covid19.who.int.

[2] Chen N, Zhou M, Dong X et al. Epidemiological and clinical characteristics of 99 cases of 2019 novel coronavirus pneumonia in Wuhan, China: a descriptive study. Lancet, 2020, 39510223: 507. 10.1016/S0140-6736(20)30211-7 PMC7135076 32007143

[3] Thunders M. and B. Delahunt Gene of the month: TMPRSS2 (transmembrane serine protease 2). J Clin Pathol, 2020, 7312: 773. 10.1136/jclinpath-2020-206987 32873700

[4] Walls AC, Park YJ, Tortorici MA et al. Structure, function, and antigenicity of the SARS-CoV-2 Spike glycoprotein. Cell, 2020, 1812: 281. 10.1016/j.cell.2020.02.058 PMC7102599 32155444

[5] Younis JS, Skorecki K (2021). Abassi the double Edge Sword of Testosterone’s role in the COVID-19 pandemic. Front Endocrinol (Lausanne).

[6] Chen J, Jiang Q, Xia X et al. Individual variation of the SARS-CoV-2 receptor ACE2 gene expression and regulation. Aging Cell, 2020, 197. 10.1111/acel.13168 PMC7323071 32558150

[7] Imai Y, Kuba K, Rao S et al. Angiotensin-converting enzyme 2 protects from severe acute lung failure. Nature, 2005, 4367047: 112. 10.1038/nature03712 PMC7094998 16001071

[8] Wang K, Gheblawi M. and G.Y. Oudit Angiotensin converting enzyme 2: a double-edged Sword. Circulation, 2020, 1425: 426. 10.1161/CIRCULATIONAHA.120.047049 32213097

[9] Romejko K, Rymarz A, Sadownik H (2022). Testosterone Deficiency as one of the Major Endocrine Disorders in chronic kidney disease. Nutrients.

[10] Çayan S, Uğuz M, Saylam B et al. Effect of serum total testosterone and its relationship with other laboratory parameters on the prognosis of coronavirus disease 2019 (COVID-19) in SARS-CoV-2 infected male patients: a cohort study. Aging Male, 2020, 23[5]: 1493. 10.1080/13685538.2020.1807930 32883151

[11] Infante M, Pieri M, Lupisella S et al. Low testosterone levels and high estradiol to testosterone ratio are associated with hyperinflammatory state and mortality in hospitalized men with COVID-19. Eur Rev Med Pharmacol Sci, 2021, 25[19]: 5889. 10.26355/eurrev_202110_26865 34661247

[12] Salonia A, Pontillo M, Capogrosso P et al. Severely low testosterone in males with COVID-19: a case-control study. Andrology, 2021, 94: 1043. 10.1111/andr.12993 PMC8013327 33635589

[13] Schroeder M, Schaumburg B, Mueller Z et al. High estradiol and low testosterone levels are associated with critical illness in male but not in female COVID-19 patients: a retrospective cohort study. Emerg Microbes Infect, 2021, 10[1]: 1807. 10.1080/22221751.2021.1969869 PMC8451658 34402750

[14] Cinislioglu AE, Cinislioglu N, Demirdogen SO et al. The relationship of serum testosterone levels with the clinical course and prognosis of COVID-19 disease in male patients: a prospective study. Andrology, 2022, 10[1]: 24. 10.1111/andr.13081 PMC8444851 34288536

[15] Toscano-Guerra E, Martínez-Gallo M, Arrese-Muñoz I et al. Recovery of serum testosterone levels is an accurate predictor of survival from COVID-19 in male patients. BMC Med, 2022, 20[1]: 129. 10.1186/s12916-022-02345-w PMC8963401 35351135

[16] Zheng S, Zou Q, Zhang D (2022). Serum level of testosterone predicts disease severity of male COVID-19 patients and is related to T-cell immune modulation by transcriptome analysis. Clin Chim Acta.

[17] Karkin K. and E. Alma Erectile dysfunction and testosterone levels prior to COVID-19 disease: what is the relationship? Arch Ital Urol Androl, 2021, 934: 460. 10.4081/aiua.2021.4.460 34933531

[18] Apaydin T, Dashdamirova S, Yazan CD et al. The association of free testosterone levels with coronavirus disease 2019. ANDROLOGY, 2022, 10[6]: 1038. 10.1111/andr.13152 34994082

[19] Moher D, Liberati A, Tetzlaff J (2009). Preferred reporting items for systematic reviews and meta-analyses: the PRISMA statement. BMJ.

[20] Wells GA, O’Connell BSD, Peterson J, Welch V, Losos M. P Tugwell. The Newcastle-Ottawa Scale (NOS) for assessing the quality of non-randomised studies in meta-analyses [Internet]. September 12, 2022]; Available from: Available at: http://www.ohri.ca/programs/clinical_epidemiology/oxford.asp.

[21] Salciccia S, Del Giudice F, Gentile V et al. Interplay between male testosterone levels and the risk for subsequent invasive respiratory assistance among COVID-19 patients at hospital admission. Endocrine, 2020, 70[2]: 206. 10.1007/s12020-020-02515-x PMC7543668 33030665

[22] Camici M, Zuppi P, Lorenzini P (2021). Role of testosterone in SARS-CoV-2 infection: a key pathogenic factor and a biomarker for severe pneumonia. Int J Infect Dis.

[23] Salciccia S, Eisenberg ML, Maggi M et al. Modeling the contribution of male testosterone levels to the duration of positive COVID testing among hospitalized male COVID-19 patients. Diagnostics (Basel), 2021, 114. 10.3390/diagnostics11040581 PMC8063957 33804969

[24] van Zeggeren IE, Boelen A, van de Beek D et al. Sex steroid hormones are associated with mortality in COVID-19 patients level of sex hormones in severe COVID-19. Medicine, 2021, 10034. 10.1097/MD.0000000000027072 PMC8389969 34449505

[25] Marinelli L, Beccuti G, Zavattaro M et al. Testosterone as a biomarker of adverse clinical outcomes in SARS-CoV-2 Pneumonia. Biomedicines, 2022, 104. 10.3390/biomedicines10040820 PMC9025790 35453570

[26] Vena W, Pizzocaro A, Maida G et al. Low testosterone predicts hypoxemic respiratory insufficiency and mortality in patients with COVID-19 disease: another piece in the COVID puzzle. J Endocrinol Invest, 2022, 45[4]: 753. 10.1007/s40618-021-01700-7 PMC8600346 34792796

[27] Yeap BB, Marriott RJ, Manning L et al. Higher premorbid serum testosterone predicts COVID-19-related mortality risk in men. Eur J Endocrinol, 2022, 1871: 159. 10.1530/EJE-22-0104 PMC9175556 35536887

[28] Montrucchio G, Corcione S, Lupia T et al. The Burden of Carbapenem-Resistant Acinetobacter baumannii in ICU COVID-19 patients: a Regional experience. J Clin Med, 2022, 1117. 10.3390/jcm11175208 PMC9456723 36079137

[29] Petrilli CM, Jones SA, Yang J (2020). Factors associated with hospital admission and critical illness among 5279 people with coronavirus disease 2019 in New York City: prospective cohort study. BMJ.

[30] Manson JJ, Crooks C, Naja M et al. COVID-19-associated hyperinflammation and escalation of patient care: a retrospective longitudinal cohort study. Lancet Rheumatol, 2020, 2[10]: e594. 10.1016/S2665-9913(20)30275-7 PMC7442426 32864628

[31] Tan L, Wang Q, Zhang D et al. Lymphopenia predicts disease severity of COVID-19: a descriptive and predictive study. Signal Transduct Target Ther, 2020, 51: 33. 10.1038/s41392-020-0148-4 PMC7100419 32296069

[32] Wang D, Hu B, Hu C et al. Clinical characteristics of 138 hospitalized patients with 2019 Novel Coronavirus-Infected pneumonia in Wuhan, China. JAMA, 2020, 32311: 1061. 10.1001/jama.2020.1585 PMC7042881 32031570

[33] Welén K, Rosendal E, Gisslén M et al. A phase 2 trial of the Effect of Antiandrogen Therapy on COVID-19 outcome: no evidence of Benefit, supported by Epidemiology and in Vitro Data. Eur Urol, 2022, 813: 285. 10.1016/j.eururo.2021.12.013 PMC8673828 34980495

